# “Double culturedness”: the “capital” of Inuit nurses

**DOI:** 10.3402/ijch.v72i0.21266

**Published:** 2013-08-05

**Authors:** Helle Møller

**Affiliations:** Department of Health Sciences, Lakehead University, Thunder Bay, Canada

**Keywords:** Greenland, Nunavut, nursing, nursing education, capital, habitus

## Abstract

**Background:**

The health and educational systems in Greenland and Nunavut are reflections of those in Denmark and Southern Canada, with the language of instruction and practise being Danish and English. This places specific demands on Inuit studying nursing.

**Objective:**

This paper discusses the experiences of Inuit who are educated in nursing programmes and practise in healthcare systems located in the Arctic but dominated by EuroCanadian and Danish culture and language.

**Design:**

Research was qualitative and ethnographic. It was conducted through 12 months of fieldwork in 5 Greenlandic and 2 Nunavut communities.

**Methods:**

Observation, participant observation, interviews, questionnaires and document review were used. The analytical framework involved Bourdieu's concepts of *capital* and *habitus*.

**Results:**

Participants experienced degrees of success and well-being in the educational systems that are afforded to few other Canadian and Greenlandic Inuit. This success appeared to be based on nurses and students possessing, or having acquired, what I call “double culturedness”; this makes them able to communicate in at least 2 languages and cultures, including the ability to understand, negotiate and interact, using at least 2 ways of being in the world and 2 ways of learning and teaching.

**Conclusion:**

There continues to be a critical need for Inuit nurses with their special knowledge and abilities in the healthcare systems of the Arctic. Inuit nurses’ experiences will help inform the education and healthcare systems and point to areas in need of support and change in order to increase recruitment and retention of nursing students and practitioners.

Nunavut and Greenland cover vast Arctic areas and have very small populations – 33,697 and 56,810, respectively (1,2) – of whom about 85% are Inuit and about 85% speak an Inuit language as their mother tongue; both have limited infrastructure with towns and settlements connected mainly by air; both were colonised by people of European descent and share social, health and education challenges similar to many other previously colonised places.

Differences exist between EuroCanadians and Inuit and between Danes and Greenlanders, in views on health, disease, healing and healthcare (3,4), as well as preferences in pedagogy and learning methods (5,6). This affects the experiences of Greenlandic and Inuit nursing students and nurses who are educated and practise in Greenland and Nunavut. The healthcare and educational systems are, in form and content, reflections of those in Denmark and Southern Canada; they function mostly in Danish and English, and are dominated by Danes and Euro-Canadians. Recruiting and retaining health professionals, especially nurses (7,8), is difficult, with almost 50% of nursing positions in the national hospital in Greenland unstaffed in 2009 (9) and 45% of all nursing positions in Nunavut vacant in 2008 (10). The nursing programmes established in Greenland in 1993 and Nunavut in 1999 to address the shortage have also had difficulties with recruitment and retention (Ruth Bainbridge (Personal Communication, Ruth Bainbridge, then Director of the Nunavut Arctic College Nursing Program, February 22, 2008). Lack of cultural appropriateness, some students suggested, may have been partly to blame (4,11).

This paper describes nurses’ and nursing students’ experiences in school from the primary level to the nursing education itself. It shows how individual, social and societal contexts, including the educational system, facilitate or hinder educational success, pointing to current strengths and needed reforms. For participant anonymity and ease of reading, unless differentiation is necessary *Inuit* is used to denote Canadian and Greenlandic Inuit, *Southerners* for EuroCanadians and Danes, *Southern language* to mean Danish or English and *Inuit language* to mean Greenlandic or Inuktitut. *European* and *Indigenous* are used for populations not covered by these terms.

Very few authors have portrayed the experiences of Inuit nurses and nursing students. This paper draws on recent work to show that reported cultural tensions motivated the current study.

In a review of Nunavut Arctic College, students noted that curriculum placed too much emphasis on Southern models and pedagogy; they expressed concern at the absence of Inuktitut in the nursing programme (12). Similarly, an evaluation of the Aboriginal nursing access programme in Labrador, where part of the curriculum was delivered in Inuktitut, stressed that cultural relevance was an “important theme in the design, development, and delivery of curriculum” (13, p. 16) and should be increased. The evaluation was based on interviews with programme stakeholders.

Based on interviews about recruitment and retention of Inuit nurses in Nunavut, Aarluk wrote that one of the “critical barriers to the entry of Inuit youth into the nursing profession is the quality of education they receive in the elementary and secondary school system” (10, p. 20), including that the language of instruction is English rather than Inuktitut. Aarluk continued that the curriculum in the nursing education does not “sufficiently reflect or support Inuit culture and society” (10, p. 20).

The Danish nursing magazine *Sygeplejeskern* published a series of articles about Greenlandic nursing students that also highlighted the important role of language and culture in student participation and success (14–17), with some authors noting that students did not seem engaged and did not say much in class (16,17), and others noting that that the chosen pedagogy may not fit the Greenlandic setting (11,15,17). Some students responded that the researchers over-generalised and treated the students in a manner resembling the paternalistic way Danish researchers treated Greenlanders in the past (5). Paternalism was also a concern of a student interviewed by Møller (4), who said that some Inuit nursing students dropped out because they felt discriminated against due to the dominance of Southern language and values.

In addition, work exists that describes the experiences of Indigenous nurses and students immersed in healthcare and educational systems dominated by European culture and languages in non-Arctic contexts in Canada (18–20), Australia (21–23) and the US (24–26). Common in this work are experiences of discrimination and cultural displacement that echo those described in the literature on Inuit and other Indigenous peoples in primary and secondary school. Berger's (6) review of the literature on Inuit, Indigenous and minority schooling paints a picture of students often struggling in European-moulded education systems; current statistics on educational attainment in Greenland and Nunavut provide evidence of this continuing struggle with <25% of student cohorts completing high school in Nunavut and Greenland (27,28), although these numbers have to be read with caution.

Southern- and European-moulded educational systems generally favour students with a particular learning *habitus* that includes: being competitive (29), working individually and for individual gain (30), being vocal in class to allow the teacher to evaluate level of understanding (31), accepting teacher authority (5), accepting teacher and book knowledge as truth (32) and considering it natural for students to sit still and listen (6). Inuit learning *habitus* includes non-hierarchy between teacher and student (5), collaboration between students and teacher to determine learning conditions, and between students to facilitate learning outcomes, and a recognition of local and lived experiences as knowledge (33).

The need to recruit and retain more Inuit nurses in Nunavut and Greenland, and the literature describing cultural tensions, suggested the need to look at the experiences of Inuit nurses and nursing students in greater depth.

## Materials and methods

The research was approved by the University of Alberta Research Ethics Board, Nunavut Research Institute, and the Research Ethics Board for Health Science Research in Greenland. It was conducted in 5 Greenlandic and 2 Nunavut communities under an anthropological frame, with 12 months of fieldwork between August 2007 and June 2009. The methods used to conduct the research included in-depth interviews and questionnaires, observation, participant observation and reviews of news reports, government documents and reports from Inuit organisations. Bourdieu's (34) concepts of *capital* and *habitus*, discussed below, were used in the analysis.

## Interviews and questionnaires

In order to include the greatest number of nurses and nursing students, the possibility of being interviewed or answering an in-depth questionnaire was offered. All nurses and students who were interviewed or who completed questionnaires were Inuit. In Greenland, 11 students and 12 nurses were interviewed and questionnaires were received from 7 students and 2 nurses. In Nunavut, 6 students and 11 nurses were interviewed (of whom 2 were employed in another Inuit region), all of whom actively practised or studied nursing. At the time of research, the sample represented about 30% of the nursing students in Greenland, 75% of Inuit nursing students in Nunavut, 20% of Greenlandic nurses and 70% of Nunavut's Inuit nurses. In order to contextualise data from active nurses and students, questionnaires were received from 8 students in Greenland and 1 in Nunavut who had opted out of the programme.

The interview-guide (35) included questions such as: tell me about your experiences in elementary school; tell me about your experiences in the nursing programme. Additional questions focused on experiences with teachers, pedagogy and language. Questionnaires contained the same questions as the interview-guide, with open-ended questions having additional prompts. Interviews lasted from 1 to 3 hours and were audio-recorded.

To help eliminate bias, the initial analysis was completed while in the communities and was shared with participants to allow participant comment and verification (36). This approach has been recommended by the National Aboriginal Health Organization (37).

## Observation and participant observation

Observation and participant observation have been important in anthropological practise since Franz Boas’ fieldwork among Inuit on Baffin Island in the late 1800s. Boas’ use of these methods sprung from the idea that “phenomena are the result of the physical and psychical character of men (sic), and of its development under the influence of the surroundings” (38, p. 587). In the 1950s, Evans-Prichard (39), among others, confirmed the importance of observation and participant observation in anthropology, and it continues to be a cornerstone today (40). Rather than “whole societies” as was the case for Evans-Pritchard (39), the cultures, societies or communities in which we immerse ourselves today may be “subcultures” or communities of practise, such as nursing, within or across societies. Regardless of the size of the community under study, the activities taking place within it, verbal and physical, continue to be situated (41) and worthy of direct observation.

As part of participant observation, informal conversations were conducted with students, nurses and other healthcare professionals and health educators in formal and social settings. Being part of nurses’ and students’ life-worlds allowed insight into the practises and the pedagogy they preferred and utilised in different contexts and shed light on their beliefs about how knowledge is created and passed on. The conversations also highlighted the heterogeneity of culture, ways and values in Greenland and Nunavut – allowing comparison between the general population and nursing students.

## Analysis

Transcribed interviews, questionnaires and fieldnotes were read several times and individually coded during and after fieldwork. Individual codes were ordered into subthemes, themes and overarching themes that, although distinct, also overlap. Four themes that emerged in relation to educational experiences include *double culturedness, language across cultures*, *body language and learning preferences*, all of which are part of our *habitus*. These themes collectively make up the overarching theme of *Embodied Cultural Capital* as depicted in Table I.

**Table I:** Themes emerging through analysis

Example of quote	Sub-theme *Habitus*	Theme	Overarching theme
*a) It is tremendously difficult to translate those theories to an Inuit language … I believe that we work with 2 things in our brains simultaneously … I understand this theory in a Southern language, I am not able to translate it into an Inuit language … Although I act on it as an Inuk, I just have the Southern understanding with me all the time (T32:185)*.	a) Bilingualism	Double-culturedness	Embodied cultural capital
*b) My parents were with the Southern teachers and principals a lot and were accepted among them and I probably learned Southern ways through that interaction. That may be why I feel very comfortable in both cultures (T48:30)*.	b) Bi-ethnicity		
*a) When I speak an Inuit language then we can speak professionally but we can also revert to something more intimate (T30:395)*.	a) Inuit use and mastery of Inuit language	Language across cultures	Embodied cultural capital
*b) When I speak a Southern language I talk about something I have learned … through my education, professional language. Something that is tied to something rational (T30:395)*.	b) Inuit use and mastery of Southern language		
*a) It is not always that my patients need to say anything. By looking at them one can see how they feel (T20:193)*.	a) Inuit use and mastery of Inuit body language	Body language	Embodied cultural capital
*b) The body language is very important. Many Southerners think that if we move our eyebrows up and down a little it means something kinky, but that is not it. It is a way of communicating (T29:310)*.	b) Southern Use and mastery of Inuit body language		
*a) The Inuk teacher does it in a way so that we are all equal. An Inuk teacher does not act as if he or she is the teacher, something special, an authority* (T6:211).	a) Inuit pedagogy/ learning	Learning/pedagogical preferences	Embodied cultural capital
b) *This Southern teacher, she always has a program for the day … everything is very scheduled. That is difficult, because you can't always follow the schedule* (T7:379).	b) Southern pedagogy/learning		

## Themes and discussion

The choice of using Bourdieu's (34,42) concepts of *habitus* and *capital* emerged while working with the material and discussing preliminary findings with nurses and students. Two general systems of thinking and actions were prominent in the data, one that was Southern and one that was Inuit. Although the Southern system dominates the educational and healthcare systems in the Arctic, both the Southern system and the Inuit System could, according to nurses and students, work as a facilitator (or *capital* as described by Bourdieu [34]) – or as a barrier – to the student or nurse.

Capital, according to Bourdieu, comes in 3 forms: economic, cultural and social; cultural capital is most relevant for this paper. Cultural capital also exists in 3 forms, an embodied, an objectified and an institutionalised. The embodied form, which is the particular disposition of our mind and body including our language (34), is the focus here. Bourdieu (42) explains that our cultural capital or “social assets” come from *habitus*, the development of which starts in infancy and which generally stays with us throughout our lives, though changes in *habitus* are possible. *Habitus* guides how we express ourselves in the world. It guides how we view and classify the world; it affects how we communicate, act and practise. Our *habitus* can provide capital or it can be a liability depending on where we are and with whom. Generally, we relate more easily to those with whom we share more *habitus*. Based on this brief discussion of *habitus* and capital, the concepts as they relate to the nurses’ and students’ experiences are discussed below.

## Embodied cultural capital

### Double culturedness

The term *double culturedness* arose from the statement: “you need to be double-cultured to function here”, which was reiterated in many different ways by many nurses and students. Double culturedness implies that nurses and students felt at home in both the Southern and the Inuit culture, which included that they were able to communicate in at least 2 languages and cultures, and understand, negotiate and interact, using at least 2 ways of being in the world and 2 ways of learning and teaching. In other words, double culturedness was seen as something positive; competence in both the Southern and the Inuit cultures or systems “gives access to” – what characterises capital in a Bourdieuian sense (34).

The following statistics are based on the 49 interviews and questionnaires as described above. Between 85 and 90% of participants were most comfortable reading, writing and being taught in a Southern language, and a much higher percentage than the average Greenlandic and Nunavummiut populations (43) spoke only a Southern language, or spoke a Southern language more than an Inuit language, in their current home (50% of Greenlandic and 65% of Canadian Inuit participants vs. 7% of Greenlanders and 8% of Nunavummiut). Twenty-one percent of Greenlandic and 38% of Canadian Inuit participants thought it would be desirable for the nursing education to eventually be delivered in an Inuit language. When asked whether they thought it would be better to be taught by Inuit or Southern teachers, 24% answered Inuit and 40% Southern, while 36% said it did not matter as long as the teacher was capable and knowledgeable.

These statistics fit with the fact that many more participants than the general Inuit populations have grown up with 1 non-Inuit parent (43) and that many more participants than the average Greenlandic and Nunavummiut populations had a non-Inuit partner (43). These factors may partly explain why Inuit nurses and students have experienced degrees of success in the educational institutions they have attended that many other Inuit have not (6). All but a few expressed that they had enjoyed public school, were good students who achieved high grades, and generally got along well with their teachers.

It is likely that the educational experiences of Inuit nurses and students have been positive because they have possessed or acquired cultural capital or *habitus* that “fits” the system – a system that catered, and continues to cater, to Southern ways and values. In many different ways, Inuit nurses and students demonstrated that they shared more *habitus* with Southerners than many other Inuit do. Despite possessing this capital, Inuit nurses and students still noted differences in *habitus* between themselves and their teachers, and sometimes between themselves and other Inuit. While these differences sometimes negatively impacted participants as elementary school, high school and university students, it appears to have been to a much lesser degree than with other Inuit.

### Education across languages

While they had close Southern relations, strong linguistic abilities and positive educational experiences, many participants noted that they sometimes found it difficult to express themselves in the Southern language in a way that would make their Southern nursing teachers understand, and that the written language also posed problems. One participant said: *Sometimes [in class] when we students thought about something in an Inuit language and were trying to express it in a Southern language … it did not work, because we were not able to translate our thoughts* (T26:152). Another participant said: *I thought that since I spoke a Southern language, I could also write in [it]. But after I started I realised that … I was not able to properly express myself in a Southern language in writing* (T20:18).

Many said that peers who mostly spoke an Inuit language had often struggled in school, had not graduated from high school, and would likely have difficulty finding a career. Students from Nunavut said that the individuals who dropped out of the nursing programme often had difficulty with the Southern language, and conversations with Greenlandic students in the health assistant programme revealed that their Southern language abilities prevented them from pursuing a nursing education, even though they had good grades in elementary and high school.

Hansen argued that the ways in which teaching is structured in the current Greenlandic educational system is fundamentally different from the learning *habitus* of most Inuit students (5). This is also true in the Canadian Arctic, where T. Berger (44) argues for a restructuring of Nunavut's educational system because of linguistic and cultural differences, recommending a system that is bilingual and bicultural in order for recruitment, retention and graduation rates to improve.

Despite seeing their Southern language ability as an asset, the vast majority of participants wanted to learn anatomy, physiology and medical terminology in an Inuit language. Some felt they needed an interpreter to properly address some things with their patients, and several indicated the desire for an Inuktitut language medical terminology course.

Most participants had just accepted that they were educated in a Southern language in elementary and high schools, and more than two-thirds had, as noted, no desire for the nursing education to be taught in an Inuit language. This might be a result of their having had a Southern parent or other significant Southern influences in their up-bringing, so it felt “normal” to speak both a Southern and an Inuit language, even though doing so also offered some challenges on their educational journey. It may also be a result of avoiding conflict, as noted by 1 nurse when discussing why Inuit, herself included, did not discuss how the heavy Southern presence in education and healthcare had affected Arctic social relations, culture and language. She said: *I have a tendency to be overly considerate of people from the South and maybe thankful that they are here and have helped us, and maybe I do not really like to criticize* (T32:221).

As a Danish researcher living in southern Canada, this reticence to criticise Southerners may have impacted findings.

Many participants expressed the pragmatic view that since they would always need Southern staff, and since developing and printing educational material in an Inuit language would be extremely costly, speaking a Southern language made sense. But whereas possessing this background and these views may be the reality for many nursing students and practising nurses, this does not reflect the reality of many Inuit who have less exposure to Southern language and culture and who do not finish elementary school, high school or post secondary education. They may have been pushed out by language and cultural demands, rather than by lack of ability or aptitude.

### Body language

Language is more than the spoken and written word; it also comprises facial expressions, gestures and movements, which all fall in the realm of Bourdieu's embodied cultural capital or *habitus* (33,42). Many participants mentioned the importance of body language in the teaching situation. One Inuk teacher said that she used body language as a means to ask a particular student to participate in class without asking the student verbally. This way, she said, she honoured an Inuit trait of non-confrontation. A student discussed how she read from the body language of an Inuk mentor to determine whether she was on the right track, whereas she would generally not be able to read anything from a Southerner.

Although the Inuit nursing students have the *embodied cultural capital* of bilingualism, the body language, which constitutes *embodied cultural capital* among Inuit may not be capital (an advantage) in instances where the mentor is Southern. Bourdieu (42) did note, however, that when people are aware that their *habitus* is not affording them success it is possible to change – as Inuit students and some long-term Southern teachers do.

### Learning/pedagogical preferences

Most participants said that many Southern teachers who had lived in the north for longer periods had learned about local ways of communicating and interacting, and had been supportive and encouraging. Still, many also expressed that some teachers displayed little knowledge about Inuit culture or history. As stated by one student: *I think it is very important [that the teacher has] cultural knowledge. If the teacher does not … we students sense it and then not many of us pay attention* (T16:88).

Other aspects of teacher attitudes and pedagogy that were highlighted included: the need for teachers to treat students with respect; allowing learning to be interactive; giving students time and opportunity to participate with “real” input, rather than just answer questions posed by the teacher or regurgitate what they had read; and that teachers be flexible and process-oriented rather than time-oriented. One student noted: *Sometimes a Southerner explains all sorts of things that are not necessary, because we know already. The Inuk teacher does it in a way so that we are all equal. An Inuk teacher does not act as if he or she is the teacher, something special, an authority* (T6:211). Another student said: *This Southern teacher, she always has a program for the day …. At this time we will do this, at this time we will do that* (T7:379).

The student added that it would be stressful as it was not always possible to keep to the schedule and both students and teachers would be more focused on time than the topic.

However, because Inuit nurses and students are double-cultured, they are able to negotiate the cultural gaps between Inuit and Southerners, including language, body language and differences in pedagogical approaches. They graduate in the existing Arctic nursing programmes – and after graduation they adapt their nursing to fit the context where they practise, as do other Indigenous nurses who are educated in European-moulded educational and healthcare systems (21,23). This is an enormous strength, as they negotiate Southern colleagues and systems while understanding Inuit patients. These findings are discussed elsewhere (45,46).

## Conclusion

Arctic nurses and nursing students are among few Inuit who possess the linguistic and cultural capital required to excel in the Southern framed educational systems that have prevailed in the Arctic during their elementary, secondary and postsecondary education. Despite efforts at reform, the Southern frame persists. This is not a problem *per se* for the nurses who graduate or for the healthcare system in which they are employed (except that there are not enough of them). It is of concern, however, that Inuit who do not possess a high amount of Southern linguistic and cultural capital are unable to excel in the educational systems, although they may have both the desire and ability to learn. For this to change, the education systems must change (5,44).

In Greenland and Nunavut, restructuring of the educational systems is underway, though change is slow. Nunavut is now offering a course in Inuktitut medical terminology in the nursing programme and has developed a new medical terminology dictionary (personal communication, Director of the Nunavut Arctic College Health and Wellness Programs Judith Paradis-Pastori, May 7, 2012), and in the Greenlandic nursing programme, the development of Greenlandic material is being considered (personal communication, Director of Nursing Ella Skifte, September 27, 2012). Time will tell what the outcome will be and what restructured educational systems will mean for prospective nursing students, some of whom may not be able to enter the programmes currently because their linguistic and cultural capital is not the sort that the educational system demands. In the meantime, with their double culturedness, Inuit nurses bring great strength to the healthcare systems, and many more Inuit nurses are needed.
